# Inhibition of Endocannabinoid Metabolism by the Metabolites of Ibuprofen and Flurbiprofen

**DOI:** 10.1371/journal.pone.0103589

**Published:** 2014-07-25

**Authors:** Jessica Karlsson, Christopher J. Fowler

**Affiliations:** Department of Pharmacology and Clinical Neuroscience, Umeå University, Umeå, Sweden; UAE University, Faculty of Medicine & Health Sciences, United Arab Emirates

## Abstract

**Background:**

In addition to their effects upon prostaglandin synthesis, the non-steroidal anti-inflammatory drugs ibuprofen and flurbiprofen inhibit the metabolism of the endocannabinoids 2-arachidonoylglycerol (2-AG) and anandamide (AEA) by cyclooxygenase-2 (COX-2) and fatty acid amide hydrolase (FAAH), respectively. Here, we investigated whether these effects upon endocannabinoid metabolism are shared by the main metabolites of ibuprofen and flurbiprofen.

**Methodology/Principal Findings:**

COX activities were measured via changes in oxygen consumption due to oxygenation of arachidonic acid (for COX-1) and arachidonic acid and 2-AG (for COX-2). FAAH activity was quantified by measuring hydrolysis of tritium labelled AEA in rat brain homogenates. The ability of ibuprofen and flurbiprofen to inhibit COX-2-catalysed oxygenation of 2-AG at lower concentrations than the oxygenation of arachidonic acid was seen with 4′-hydroxyflurbiprofen and possibly also 3′-hydroxyibuprofen, albeit at lower potencies than the parent compounds. All ibuprofen and flurbiprofen metabolites retained the ability to inhibit FAAH in a pH-dependent manner, although the potency was lower than seen with the parent compounds.

**Conclusions/Significance:**

It is concluded that the primary metabolites of ibuprofen and flurbiprofen retain some of the properties of the parent compound with respect to inhibition of endocannabinoid metabolism. However, these effects are unlikely to contribute to the actions of the parent compounds *in vivo.*

## Introduction

Non-steroidal anti-inflammatory drugs (NSAIDs) mediate their anti-inflammatory, analgesic and antipyretic action by inhibiting production of prostaglandin intermediates by inhibition of cyclooxygenase isoenzymes 1 and/or 2 (COX-1, COX-2) [1]. NSAIDs are widely used although their ability to produce serious unwanted cardiovascular and gastrointestinal effects means that there is a need for novel compounds with an improved safety profile.

The endocannabinoid system has been implicated in the mechanism of NSAIDs (reviewed in [2]). The endocannabinoids anandamide (arachidonoylethanolamide, AEA) and 2-arachidonoylglycerol (2-AG) are substrates for COX-2 but not COX-1 cyclooxygenation producing prostaglandin -ethanolamides and -glycerol esters [3]–[5]. Ibuprofen is a potent noncompetitive COX-2 inhibitor of 2-AG cyclooxygenation (IC_50_∶210 nM) but a weak, competitive inhibitor of arachidonic acid (AA) oxygenation (IC_50_∶180 µM) and this property is shared by other profens such as flurbiprofen [6]–[8]. In our hands, flurbiprofen inhibits 2-AG oxygenation with an IC_50_ value of 1.3 µM. The corresponding values for inhibition of AA oxygenation by COX-1 and -2, respectively, are 3.6 and 103 µM [8]. Thus, the traditional description of flurbiprofen as a COX inhibitor with some selectivity for COX-1 over COX-2 is only true when AA is used as substrate. Mechanistically, this is because COX-2 is a dimer with half-sites reactivity. Binding of the NSAID to the allosteric site of the homodimer prevents endocannabinoid oxygenation, whereas inhibition of AA oxygenation requires NSAID binding to the catalytic site [6], [7]. Differences in affinities for the two sites account for the substrate-selectivity. More recently, an indomethacin analogue with pronounced selectivity for inhibition of COX-2 catalysed endocannabinoid oxygenation vs. AA oxygenation has been described. This compound increases endocannabinoid levels in the brain in vivo and produces potentially beneficial effects in models of anxiety [9], thus demonstrating that the ability of COX-2 to metabolise endocannabinoids is a physiologically relevant pathway, at least in the brain.

In addition to the effects upon COX isoenzymes, the profens inhibit the hydrolysis of AEA by the enzyme fatty acid amide hydrolase (FAAH) [8], [10]–[14]. Although the potencies are relatively low (e.g. 270 µM for ibuprofen [10]), they are increased 3–4-fold at low pH [15], [16]. A lowered extracellular pH (such as is seen in inflamed tissue [17]) is sufficient for this to be seen [18]. Given the ability of acidic NSAIDs to accumulate in inflamed tissue [19], it is possible that local inhibition of FAAH can contribute to the effects of the profens in such tissues. Certainly, locally administered ibuprofen synergizes with AEA in rat models of pain and inflammation [20]. The synergistic effect is antagonized by the cannabinoid CB_1_ inverse agonist AM251 suggesting inhibited AEA metabolism as the mechanism [20]. Indomethacin is acidic and accumulates in inflamed tissue [19], and reduces carrageenan-induced oedema in a manner blocked by the CB_2_ receptor inverse agonist SR144528 [21], a finding consistent with a mobilization of the endocannabinoid system under these conditions.

**Figure 1:**
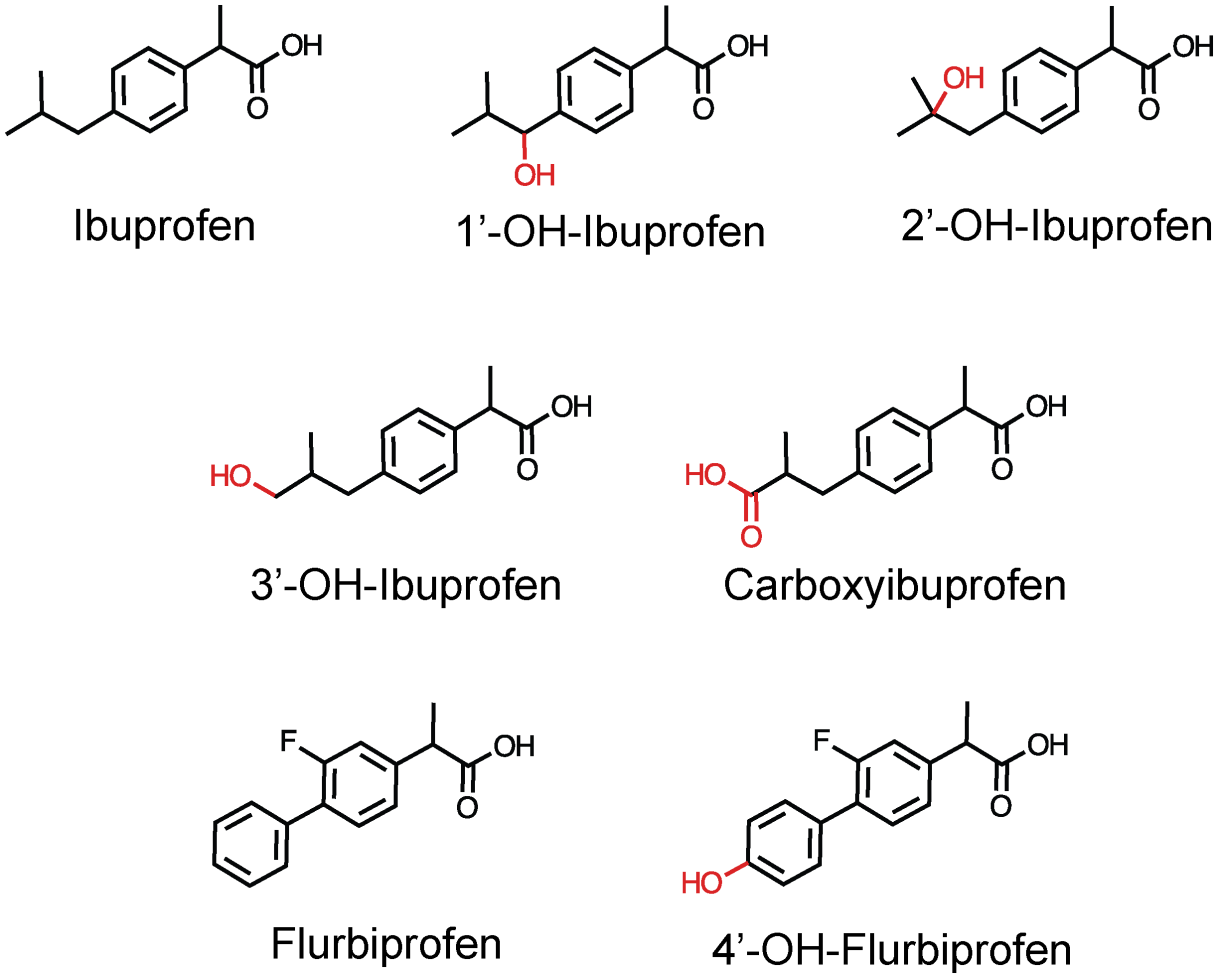
Structures of ibuprofen, flurbiprofen and the metabolites investigated in the present study.

Research into the effects of NSAIDs on the endocannabinoid system and the balance between arachidonic acid and endocannabinoid metabolism has focused entirely on the parent compounds and/or their enantiomers. The effects of their major metabolites have not been considered. This is of importance, given that many drugs have active metabolites that contribute to the pharmacological effects of the drugs *in vivo*. Ibuprofen is metabolized by phase I oxygenation to the major metabolites carboxyibuprofen, and 2′-hydroxyibuprofen [22] and minor metabolites 3′-hydroxyibuprofen and 1′-hydroxyibuprofen [23] (structures, see Fig. 1). 4′-hydroxyflurbiprofen is one of the major metabolites of flurbiprofen in rat and human [24] (structures, see Fig. 1). In theory, an active metabolite could show a useful profile, such as a potent inhibition of FAAH and/or blockade of 2-AG oxygenation, but not arachidonic acid oxygenation, by COX-2. In consequence, in the present study, we have compared the potencies of these metabolites with the parent compounds upon the substrate-selective inhibition of COX and the pH-dependent inhibition of FAAH.

## Methods

### Compounds

Ibuprofen ((±)-2-(4-Isobutylphenyl)propanoic acid), 1′-hydroxyibuprofen (2-[4-(1-hydroxy-2-methylpropyl)phenyl]propionic acid), 2′-hydroxyibuprofen (2-[4-(2-hydroxy-2-methylpropyl)phenyl]propionic acid), 3′-hydroxyibuprofen (2-[4-(3-hydroxy-2-methylpropyl)phenyl]propionic acid), carboxyibuprofen (2-[4-(2-carboxypropyl)phenyl]propionic acid), flurbiprofen ((±)-2-flouro-α-methyl(1,1′-biphenyl)-4-acetic acid) and fatty acid-free bovine serum albumin were obtained from Sigma Aldrich. (St Louis, MO, USA.). 4′-Hydroxyflurbiprofen (2-(4-hydroxy-2-fluoro-4-biphenylyl)propionic acid) was obtained from Santa Cruz Biotechnology (Heidelberg, Germany). The profens were dissolved in DMSO. Arachidonyl etanolamide-[1-^3^H] (AEA-^3^H) (specific activity of 2.2 TBq mmol^−1^) was purchased from American Radiolabeled Chemicals, Inc (St Louis, MO, USA). Ovine COX-1 (cat. no. 60100), human recombinant COX-2 (cat. no. 60122), arachidonic acid, 2-arachidonoylglycerol and non-radioactive anandamide were purchased from the Cayman Chemical Co. (Ann Arbor, MI, USA). Substrates were dissolved in ethanol.

### Preparation of rat brain homogenates

Brains (minus cerebella) from adult Wistar or Sprague-Dawley stored at −80°C were thawed, weighed and homogenized in cold buffer (20 mM HEPES, 1 mM MgCl_2_ pH 7.0). Homogenates were centrifuged at 35,000 *g* at 4°C for 20 min and the pellet was resuspended in homogenization buffer (repeated twice) before being incubated at 37°C for 15 min to degrade any endogenous substrate that might interfere with the FAAH assay. Following centrifugation, the pellets were resuspended in cold buffer (50 mM Tris-HCl, 1 mM EDTA, 3 mM MgCl_2_, pH 7.4). The protein concentration of each homogenates was determined according to the Bradford assay [25] after which the samples were frozen in aliquots at −80°C until used for FAAH assay. Ethical permission for the animal experiments was obtained from the local animal research ethical committee (Umeå Ethical Committee for Animal Research, Umeå, Sweden).

### Cyclooxygenase assays

The assay was performed according to Meade *et al.* [26] with minor modifications [27] using an oxygen electrode chamber with integral stirring (Oxygraph System, Hansatech Instruments, King’s Lynn, U.K.). The electrode was calibrated to ambient temperature and air pressure. Samples were prepared in 0.1 M Tris-HCl buffer pH 7.4 containing 1 µM haematin, 2 mM phenol, 5 mM EDTA, 10 µM substrate (AA or 2-AG) (final assay volume was 2 ml). After addition of test substance dissolved in DMSO (final assay concentration 1%) a baseline was established for 5 min before initiation of reaction by addition of 200 units COX-1 or COX-2. Change in oxygen consumption was monitored for approximately 5 min. Flurbiprofen or ibuprofen were included in each experiment as positive controls.

### Assay of FAAH hydrolysis

The assay was performed according to Boldrup *et al.* [28]. Homogenates (1 µg protein per assay) diluted in 10 mM Tris-HCl, 1 mM EDTA, pH 7.4 or pH 5.1 were mixed with test substance dissolved in DMSO (total concentration in assay 5%) at an ambient temperature of 4°C. Reactions were initiated by addition of AEA-[^3^H] (assay concentration 0.5 µM) in 10 mM Tris-HCl, 1 mM EDTA, pH 7.4 containing 10 mg ml^−1^ fatty acid-free bovine serum and incubation at 37°C which was allowed to proceed for 10 min. The DMSO concentration did not affect the observed enzyme activity under the conditions used. Blank samples contained buffer instead of homogenate and DMSO (5%). The assay volume of each sample was 200 µl. The pH values given in the text were determined by measurement at 37°C of scaled-up mixtures of homogenization and assay buffers. The reaction was terminated by addition of 400 µl activated charcoal (80 µl activated charcoal +320 µl 0.5 M HCl) and placement of samples on ice. Samples were kept in room temperature for 45–60 min before being centrifuged at 2500 rpm for 10 min. Aliquots (200 µl) of the supernatant was analyzed for tritium content by liquid scintillation with quench correction.

### Statistical Analysis

FAAH inhibition was calculated using log(inhibitor) *vs*. response with variable slope (four parameters) algorithm in the GraphPad Prism computer program (GraphPad Software Inc., San Diego, CA. USA). Data was expressed as % of control and pI_50_ and IC_50_ values where calculated from curves generated from uninhibited (top) values set at 100% and letting residual activity (bottom) values be fixed to zero or floating. The two curves were compared by Akaike’s informative criteria and the best fit was chosen. Initial COX activities were calculated as the change in oxygen tension between 10 and 30 seconds after addition of enzyme.

## Results

### Inhibition of COX cyclooxygenation by ibuprofen, flurbiprofen and their metabolites

**Figure 2:**
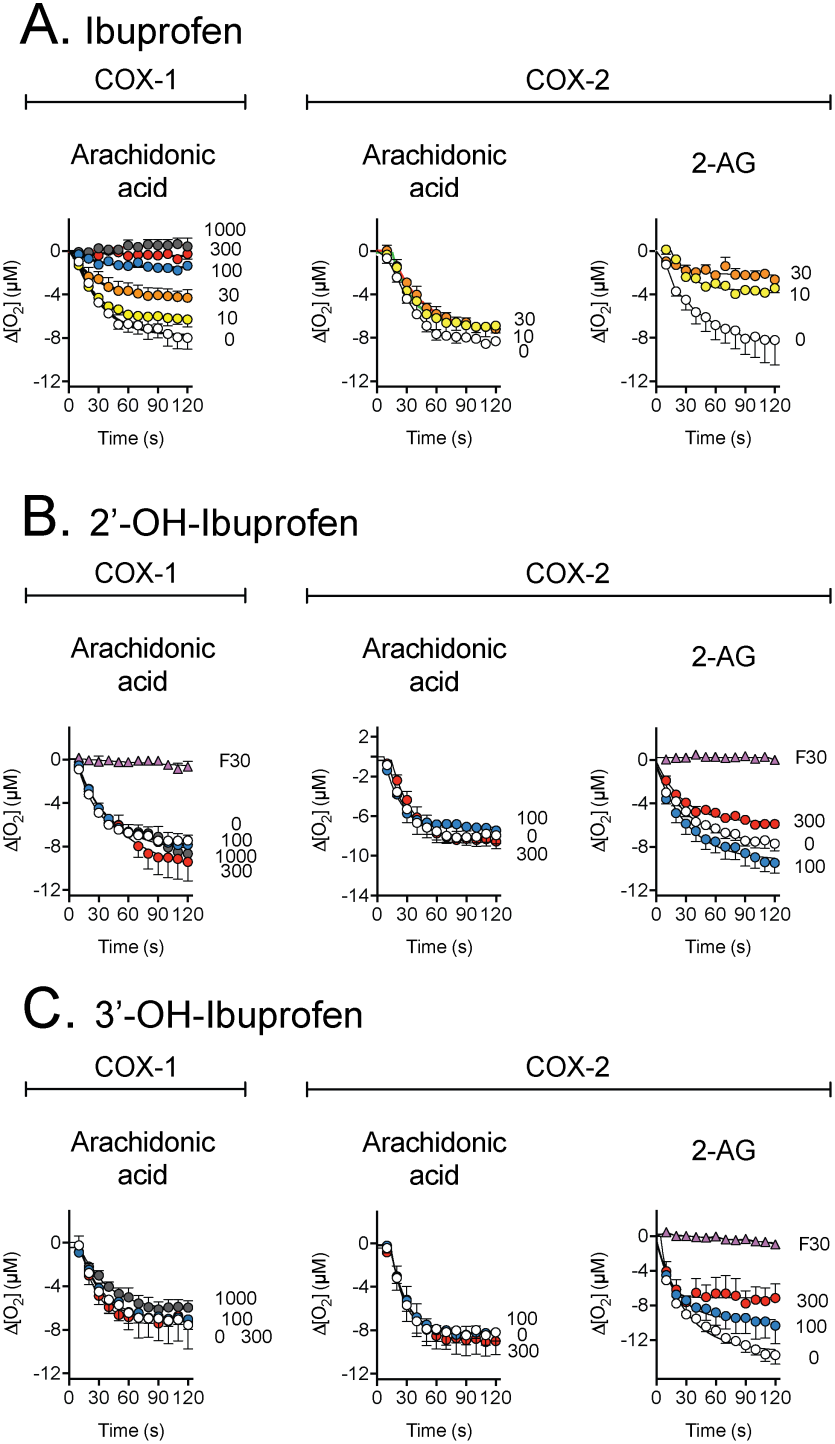
Inhibition of the activities of ovine COX-1 (towards arachidonic acid) and human recombinant COX-2 (towards either arachidonic acid or 2-AG) by ibuprofen and its 2′- and 3′-hydroxy metabolites. The substrates used (10 µM) are shown in the figure, as are the concentrations (in µM) of the test compounds. “F30” refers to 30 µM flurbiprofen, used as a positive control. Values are means ± s.e.m. (unless enclosed by the symbols), n = 3, of the change in oxygen utilisation following addition of enzyme to the oxygen electrode chamber.

**Figure 3:**
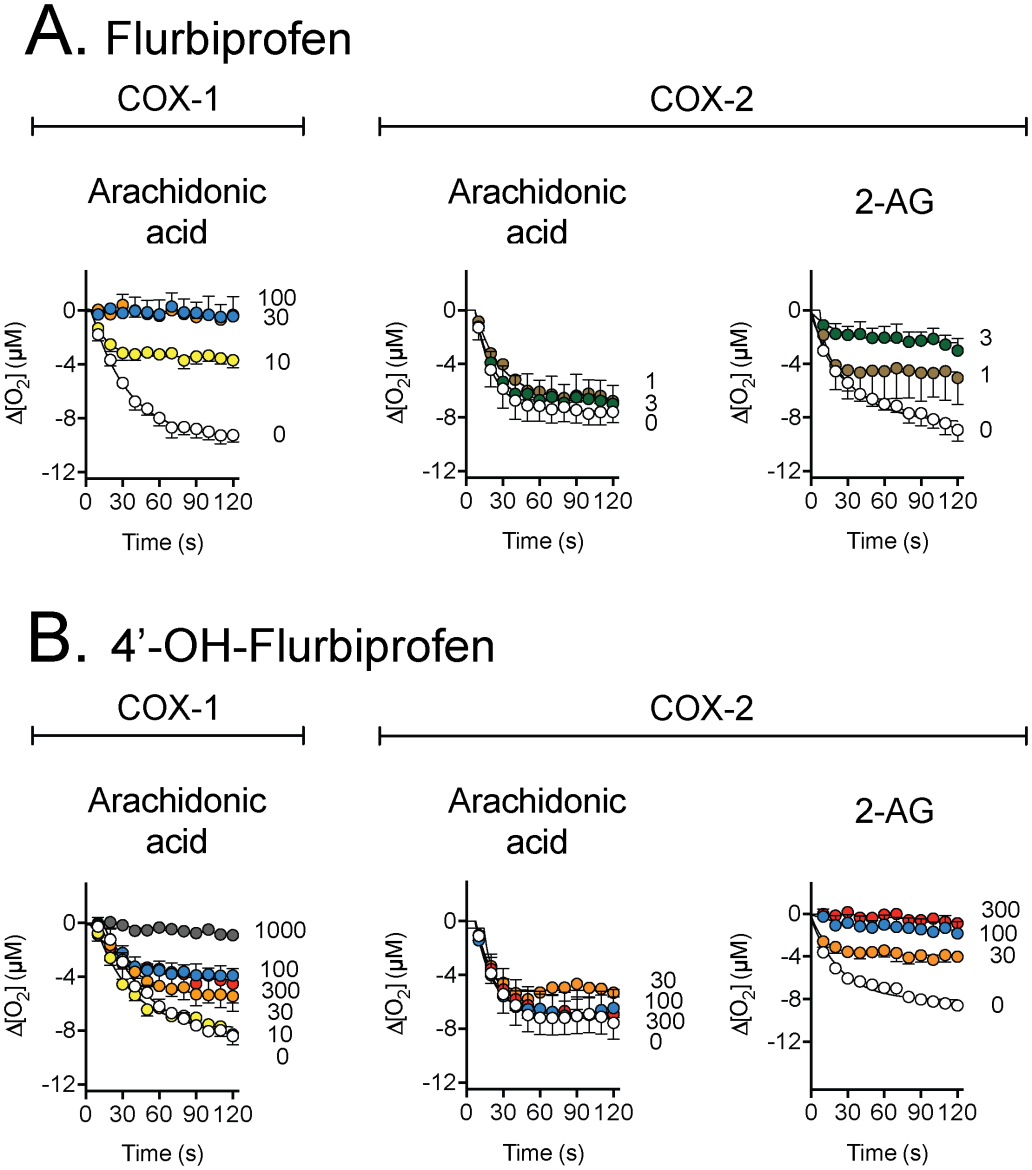
Inhibition of the activities of ovine COX-1 (towards arachidonic acid) and human recombinant COX-2 (towards either arachidonic acid or 2-AG) by flurbiprofen and its 4′-hydroxy metabolite. The substrates used (10 µM) are shown in the figure, as are the concentrations (in µM) of the test compounds. Values are means ± s.e.m. (unless enclosed by the symbols), n = 3, of the change in oxygen utilisation following addition of enzyme to the oxygen electrode chamber.

The effects of ibuprofen, flurbiprofen and their metabolites upon the activity of COX-1 (vs. AA) and COX-2 (vs. both AA and 2-AG) are shown in Figs. 2, 3, S1 and S2, and summarized in Tables 1 and 2. Activity was measured as change is oxygen concentration after initiation of reaction. As expected, 300 µM ibuprofen inhibited 98% of COX-1-catalysed oxygenation of arachidonic acid. The corresponding inhibition was 19, 9, 3 and 4%, respectively, for 1′-hydroxy-, 2′-hydroxy-, 3′-hydroxy- and carboxy- ibuprofen metabolites. Complete inhibition of COX-1 was produced by 30 µM flurbiprofen, while its metabolite 4′-hydroxyflurbiprofen produced a 3, 28 and 94% inhibition at 100, 300 and 1000 µM, respectively.

**Table 1:** Comparison of the effects of the NSAIDs and their metabolites upon the initial activity of COX-1.

	COX-1 activity (% of control)		
NSAID	100 µM	300 µM	1000 µM
Ibuprofen	26±5	2±2	−9±0.3
1′-OH-Ibuprofen	112±21	81±21	102±18
2′-OH-Ibuprofen	102±17	91±5	95±3
3′-OH-Ibuprofen	81±16	97±10	68±15
Flurbiprofen	−3±7		
4′-OH-Flurbiprofen	97±31	72±20	6±6

Initial activities were calculated as the change in oxygen tension between 10 and 30 seconds after start of reaction. The values are means ± s.e.m., n = 3.

**Table 2:** Comparison of the effects of the NSAIDs and their metabolites upon the initial activity of COX-2.

	COX-2 activity (% of control)							
[NSAID] (µM):	3		30		100		300	
	AA	2-AG	AA	2-AG	AA	2-AG	AA	2-AG
Ibuprofen			79±12	26±2				
1′-OH-Ibuprofen					91±8	103±22	59±13	108±15
2′- OH-Ibuprofen					98±17	125±24	83±12	115±21
3′-OH-Ibuprofen					103±21	73±14	84±10	91±2
Carboxyibuprofen					132±16	105±19	139±41	132±17
Flurbiprofen	89±6	30±15						
4′-OH-Flurbiprofen			88±16	39±11	106±16	24±4	106±9	−6±9

Initial activities were calculated as the change in oxygen tension between 10 and 30 seconds after start of reaction. The values are means ± s.e.m., n = 3.

Flurbiprofen (3 µM) inhibited 33% of the COX-2–catalysed oxygenation of arachidonic acid. The corresponding values for oxygenation of 2-AG at 3 µM was 70%, confirming the substrate selective inhibition of COX-2 by this compound [7], [8]. 4-Hydroxyflurbiprofen failed to inhibit COX-1 and COX-2–catalysed oxygenation of arachidonic acid, but inhibited cyclooxygenation of 2-AG completely at 300 µM and partially at concentrations of 100 µM and 30 µM, respectively. 1′-Hydroxy-, 2′-hydroxy and carboxy-ibuprofen had minor effects upon the COX isoenzymes regardless of the substrate used. However, the 3′-hydroxy- metabolite of ibuprofen retained the ability of ibuprofen to inhibit the COX-2-catalysed oxygenation of 2-AG at longer incubation times, albeit at a lower potency than the parent compound. The initial inhibition (measured between 10 and 30 s) was only modestly affected (Fig. 2, Tables 1 and 2). Preliminary data for lower concentrations of the ibuprofen metabolites towards COX-2 are shown in Fig. S2. No obvious inhibition was seen, but there was some increase in the oxygenation of the substrates, particularly with carboxyibuprofen.

### Inhibition of anandamide hydrolysis

**Figure 4:**
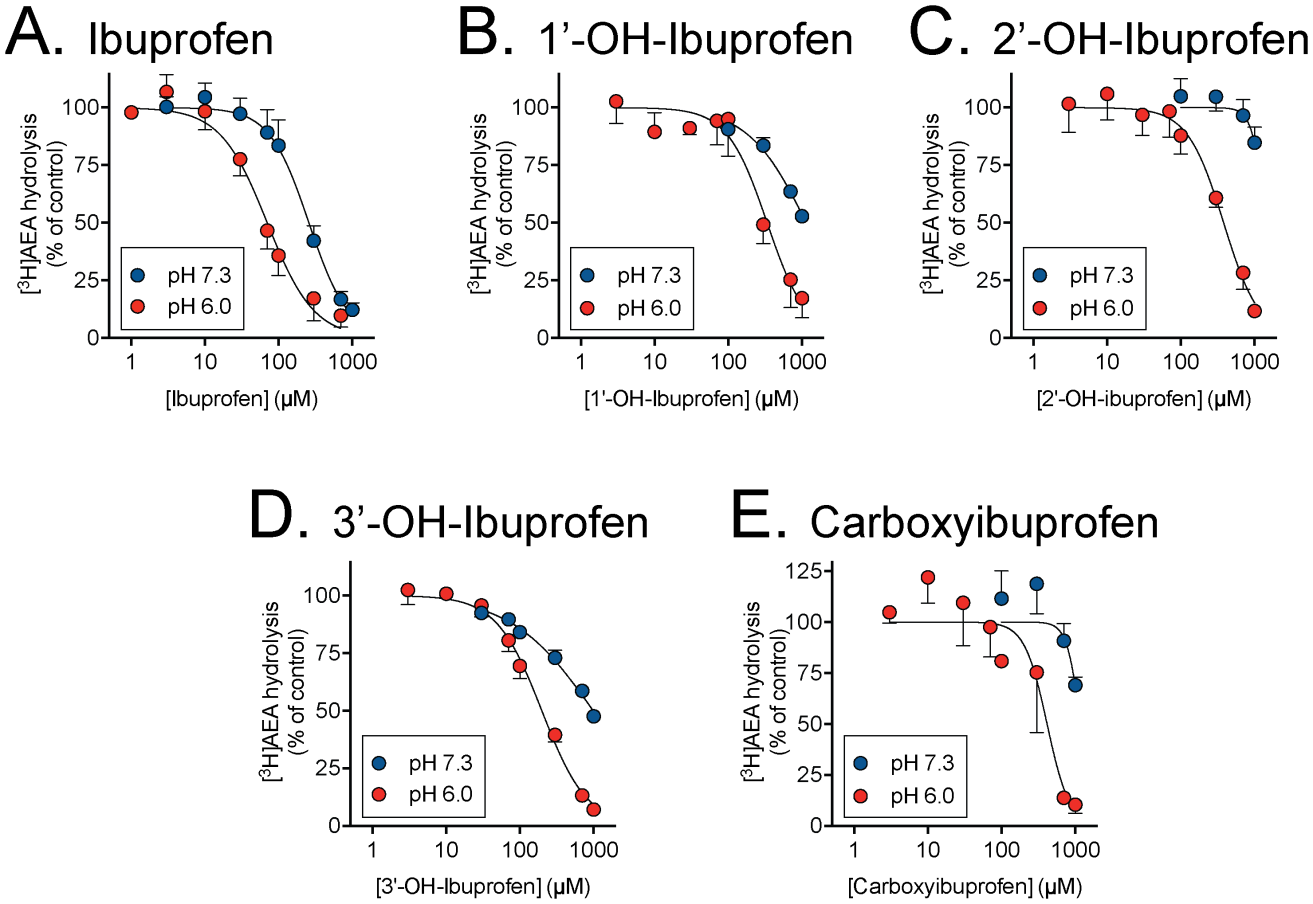
Inhibition of FAAH activity in rat brain homogenates by ibuprofen and its 1′-OH, 2′-OH, 3′-OH and carboxy metabolites. The AEA assay concentration was 0.5 µM. Values are means ± s.e.m. (unless enclosed by the symbols), n = 3.

**Figure 5:**
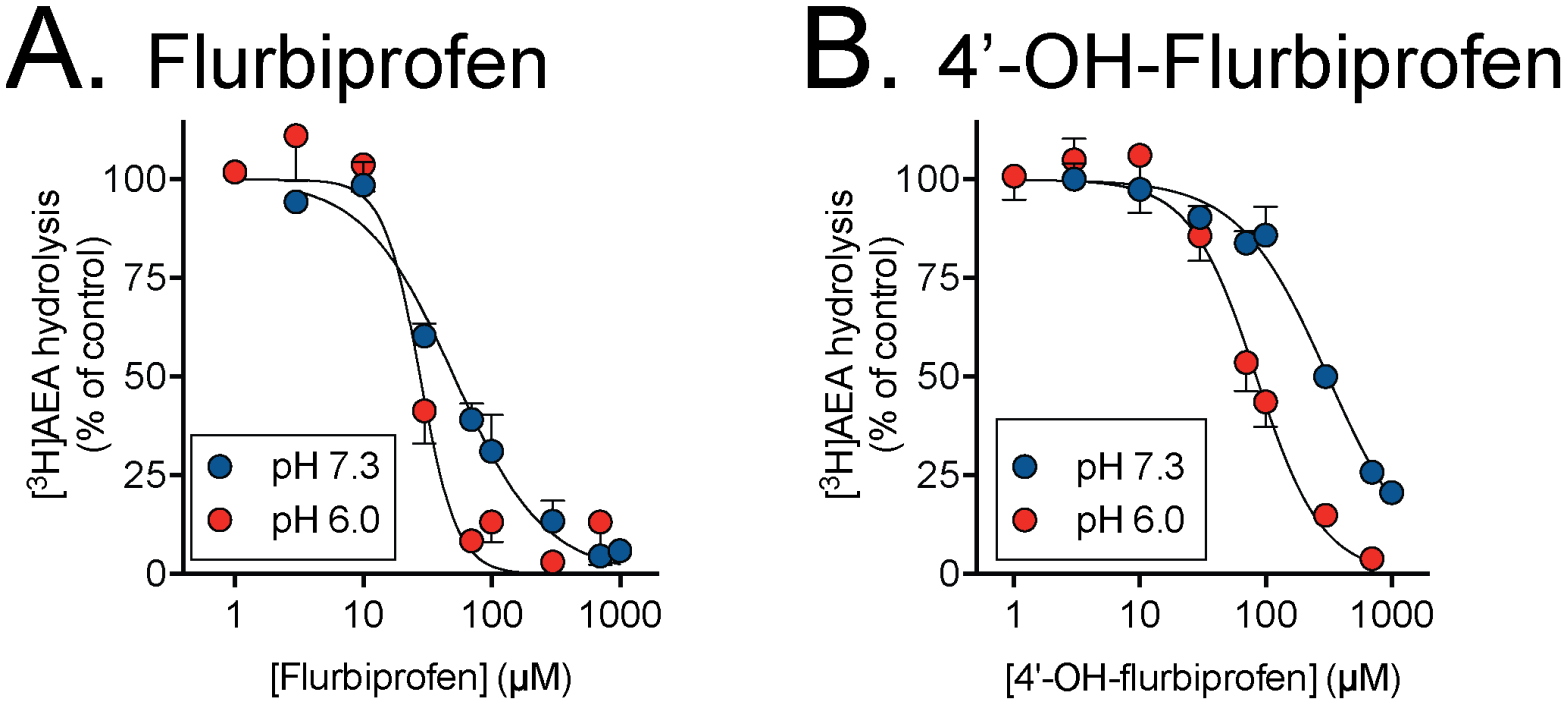
Inhibition of FAAH activity in rat brain homogenates by A, flurbiprofen and its 4′-hydroxy metabolite. The AEA assay concentration was 0.5 µM. Values are means ± s.e.m. (unless enclosed by the symbols), n = 3.

Profens inhibit anandamide hydrolysis through inhibition of the metabolic enzyme FAAH. The ability of ibuprofen, flurbiprofen and their metabolites to inhibit FAAH hydrolysis of anandamide was quantified by measuring hydrolysis of 0.5 µM tritium labelled AEA in rat brain homogenates at physiological pH 7.3 and pH 6.0 (Figs. 4 and 5). None of the metabolites were more potent FAAH inhibitors than the corresponding parent compounds. pI_50_ values are summarized in Table 3. However, they all showed a pH-dependency, with a greater potency at pH 6.0 than at pH 7.3. At the lower pH, 4′-hydroxyflurbiprofen was about three-fold less potent than the parent compound (IC_50_ values of 84 and 28 µM, respectively). The ibuprofen metabolites had IC_50_ values in the range 200–410 µM at this pH, as compared with 70 µM for the parent compound (Table 3).

**Table 3:** pI_50_ and IC_50_ values for ibuprofen, flurbiprofen and their metabolites towards the inhibition of 0.5 µM [^3^H]AEA hydrolysis by rat brain FAAH.

	pI_50_ (IC_50_, µM)	
Compound	pH 6.0	pH 7.3
Ibuprofen	4.15±0.05 (70 µM)	3.59±0.05 (260 µM)
1′-OH-Ibuprofen	3.47±0.07 (340 µM)	47±0.8% inhibition @ 1000 µM
2′-OH-Ibuprofen	3.42±0.06 (380 µM)	15±7% inhibition @ 1000 µM
3′-OH-Ibuprofen	3.70±0.03 (200 µM)	3.01±0.04 (990 µM)
Carboxyibuprofen	3.38±0.08 (410 µM)	31±4% inhibition @ 1000 µM
Flurbiprofen	4.56±0.05 (28 µM)	4.29±0.04 (51 µM)
4′-OH-Flurbiprofen	4.07±0.05 (84 µM)	3.50±0.03 (310 µM)

Values were determined using the log(inhibitor) vs. response – variable slope (four parameters) algorithm in the Prism 6 computer programe, with top and bottom values constrained to 100% and 0%, respectively. In all cases, Akaike’s informative criteria indicated that the curves were better fitted to these constraints than the equivalent algorithm where the bottom value was not constrained.

## Discussion

The major mechanism of NSAIDs is inhibition of the COX isoenzymes [1] although the ability of the profens to inhibit endocannabinoid oxygenation by COX-2 and FAAH (particularly at low pH) may contribute to their actions (see Introduction). Many drugs have active metabolites: the 6-glucuronide metabolite of morphine, for example, is 2–4 times more potent than the parent drug towards µ-opoid receptors in humans (review, see [29]). In the present study, the abilities of the main metabolites of flurbiprofen and ibuprofen to inhibit endocannabinoid metabolism by COX isoenzymes and FAAH were investigated in order to determine whether such active metabolites are also found for the profens.

Ibuprofen metabolites 1′-hydroxy-, 2′-hydroxy- and carboxy- ibuprofen failed to inhibit COX-1, and the metabolites did not show the substrate selective inhibition of COX-2 cyclooxygenation of 2-AG vs. AA displayed by the parent compound. However 4′-hydroxyflurbiprofen and possibly 3′-hydroxyibuprofen retained, albeit with a lower potency, the substrate-selective inhibition of COX-2 displayed by the parent compounds. Carboxyibuprofen appeared to increase the rate of oxygenation of 2-AG at the 300 µM concentration and AA at the 10 µM concentration (Figs. S1 and S2). This observation was not investigated further, but may reflect some sort of allosteric interaction. Palmitic acid has been shown to increase the COX-2-catalysed oxygenation of several substrates, including 2-AG, AA and adrenic acid, but not eicosadienoic acid [30] and a similar sort of event may be occurring here.

The active site of the COX-enzymes is a long, narrow, hydrophobic channel extending inwards to the core of the catalytic domain [31]. A few specific amino acids within the channel are essential for the enzymatic activity of the enzyme [31]. Flurbiprofen binds to the channel of COX-1 with its carboxyl group interacting with one of only two polar residues within the channel [31], [32]. The polar residue plays a major role in the peroxidase activity and is essential for inhibition by NSAIDs. Thus mutation of this residue reduces the affinity for AA as a substrate and disrupts inhibition by NSAIDs containing a carboxyl group [32]. 4-Hydroxyflurbiprofen is hydroxylated in position 4 of the first phenyl-group. This may add to the bulkiness of the structure and disturb ability for interaction within the narrow channel of COX-1. COX-1 and COX-2 are 67% identical with very high identity in the catalytic domain [33]. They differ in only a few residues of the active site. However, these residues cause a second pocket in the COX-2 structure very close to the NSAID binding site and may account for inhibitor selectivity [33]. Thus, 4′-hydroxyflurbiprofen can still enter the active site. COX-2 utilizes both AA and endocannabinoids as substrates, and this feature may be linked to flexibility in the active site due to rotamer conformations of leucin in position 531 not seen in the narrow channel of COX-1 [34]. Substrate selectivity for 2-AG when inhibited with 4′-hydroxyflurbiprofen and 3′-hydroxyibuprofen may arise due to half-sites reactivity that induces a conformational change limiting the flexibility in leu-531 and thus limiting the possibility for multiple substrates.

AEA is primarily hydrolysed by FAAH, an enzyme with a pH optimum in the region of 8–9 [35]. However, an *N*-acylethanolamine acid amidase with a pH optimum of 5 and capable of hydrolyzing AEA has also been described [36]. The selective FAAH inhibitor URB597 lacks activity towards NAAA [37] and is thus a good pharmacological tool to assess whether NAAH contributes to AEA hydrolysis. In rat brain homogenates assayed using the same methodology as here, the selective FAAH inhibitor URB597 completely inhibits AEA hydrolysis at both pH 6 and 8 [38] indicating that FAAH is the major contributor to AEA hydrolysis under the conditions used here. The metabolites of both ibuprofen and flurbiprofen were less potent than the parent compounds towards inhibition of FAAH, but they retained the pH-sensitivity of the parent compounds reported previously [15], [16], [18]. The results give structural insight in how changes in the profen molecule may affect the biological activity and the possibilities of this should be explored. Carprofen binds FAAH at the entrance of the active cleft with the carboxyl-group pointing outwards from the enzyme [39]. A common feature for many potent FAAH inhibitors within the family of NSAIDs are a carboxyl-group together with a phenyl ring often coupled to a chloride atom and a fused aromatic-system [39]. The phenyl group of flurbiprofen points inward the active cleft. A small change in that structure, such as the 4′-hydroxylation of the phenyl-substituent would affect how the molecule fits the active cleft and thus the ability to bind and inhibit the enzyme. Presumably similar processes can explain the loss of potency of the ibuprofen metabolites towards FAAH.

With respect to the increased potency of the compounds towards FAAH as the pH is reduced, the simplest explanation is that the non-ionised form of the compounds (at the carboxyl group, which is retained by the metabolites) are primarily responsible for the inhibition; this would certainly explain why profens are more potent as inhibitors of FAAH in intact cells when the extracellular pH is reduced [18]. However, the increase in potency as the pH is reduced is less than would have been predicted from the Henderson-Hasselbach equation. Our working hypothesis is that the observed sensitivity to inhibition is a composite of the selectivity of the non-ionised form of the profens to inhibit the enzyme coupled with a reduced sensitivity of the enzyme to inhibition per se as the pH is reduced. This is consistent with the finding that the *N*-(3-methylpyridin-2-yl)amide derivative of ibuprofen, which no longer has a carboxyl group, is in fact a slightly less potent inhibitor of FAAH at pH 6 than at pH 8 [40].

A final question concerns whether the metabolites possess sufficient potency towards FAAH (and possibly COX-2 oxygenation of 2-AG for 4′-hydroxyflurbiprofen) to contribute to the effects of the parent compounds *in vivo*. Extrapolation from *in vitro* data to the situation *in vivo* is difficult, to say the least. In the case of flurbiprofen, a dose of 50 mg produces maximal plasma concentrations of *R*- and *S*-enantiomers corresponding roughly to a concentration of racemate of 35 µM [41]. Peak plasma ibuprofen concentrations of in the range 110–150 µM have been reported after two 200 mg single doses of two different ibuprofen preparations [42]. Of course, profens bind avidly to plasma proteins, but the FAAH assays are conducted in the presence of fatty acid-free bovine serum albumin, so the conditions are reasonably close. Carboxyibuprofen is the most abundant metabolite in human plasma 3 h after a single dose of 200 mg ibuprofen [22]. Urinary recovery of carboxyibuprofen and 2′-hydroxyibuprofen compared to ibuprofen, is 40, 23 and 11%, respectively, over a period of 24 h after administration of 400 mg of ibuprofen [43]. 3′-Hydroxyibuprofen, however, belongs to the minor metabolites and is thus unlikely to reach concentrations sufficient for FAAH/COX inhibition *in vivo*. The same is presumably true for 4′-hydroxyflurbiprofen, although this metabolite accounts for 43% of the dose recovered in the urine compared to 23% for the parent flurbiprofen after a dose of 50 mg a day for 10 consecutive days [24].

In conclusion, we report that 4′-hydroxyflurbiprofen, the major metabolite of flurbiprofen inhibit FAAH and COX-2 oxygenation of 2-AG in a manner similar to the parent compound, albeit with a lower potency. Ibuprofen metabolites fail to inhibit FAAH and COX at relevant concentrations and thus are unlikely to contribute to the therapeutic effect and adverse events related to the drug.

## Supporting Information

Figure S1 **Inhibition of the activities of ovine COX-1 (towards arachidonic acid) and human recombinant COX-2 (towards either arachidonic acid or 2-AG) by 1′hydroxyibuprofen and carboxyibuprofen.** The substrates used (10 µM) are shown in the figure, as are the concentrations (in µM) of the test compounds. Values are means ± s.e.m. (unless enclosed by the symbols), n = 3, of the change in oxygen utilisation following addition of enzyme to the oxygen electrode chamber.
(TIF)

Figure S2 **Inhibition of the activities of human recombinant COX-2 (towards either arachidonic acid or 2-AG) by the metabolites of ibuprofen.** The substrates used (10 µM) are shown in the figure, as are the concentrations (in µM) of the test compounds. Values are means of two experiments of the change in oxygen utilisation following addition of enzyme to the oxygen electrode chamber.
(TIF)
