# Supplementary figures and images for: Inhibition of Endocannabinoid Metabolism by the Metabolites of Ibuprofen and Flurbiprofen
Source: PLoS One. 2014 Jul 25;9(7):e103589. doi: 10.1371/journal.pone.0103589 (PMC4111603; doi:10.1371/journal.pone.0103589)

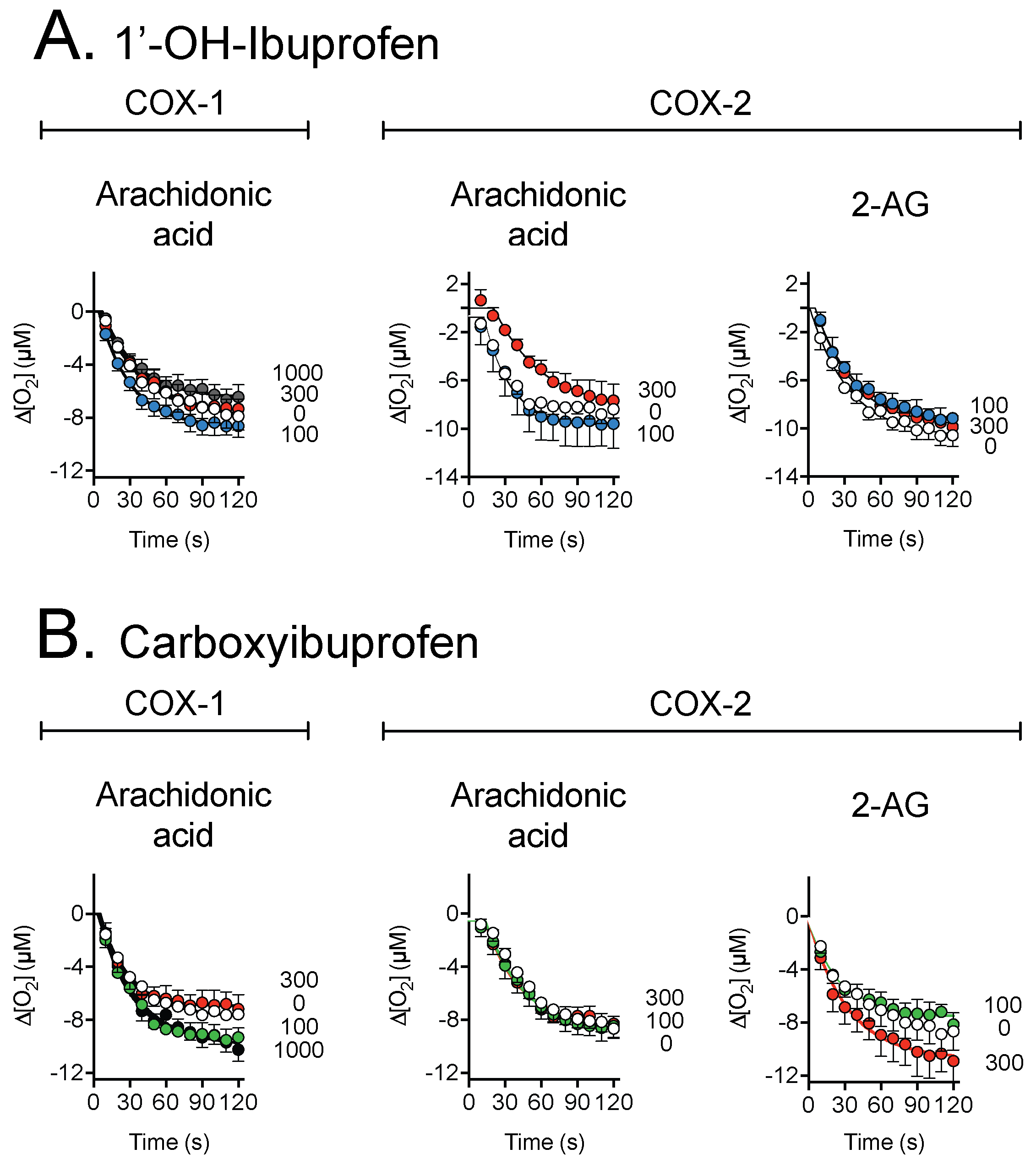

Supplement: Figure S1 — Inhibition of the activities of ovine COX-1 (towards arachidonic acid) and human recombinant COX-2 (towards either arachidonic acid or 2-AG) by 1′hydroxyibuprofen and carboxyibuprofen. The substrates used (10 µM) are shown in the figure, as are the concentrations (in µM) of the test compounds. Values are means ± s.e.m. (unless enclosed by the symbols), n = 3, of the change in oxygen utilisation following addition of enzyme to the oxygen electrode chamber. (TIF) [file pone.0103589.s001.tif]

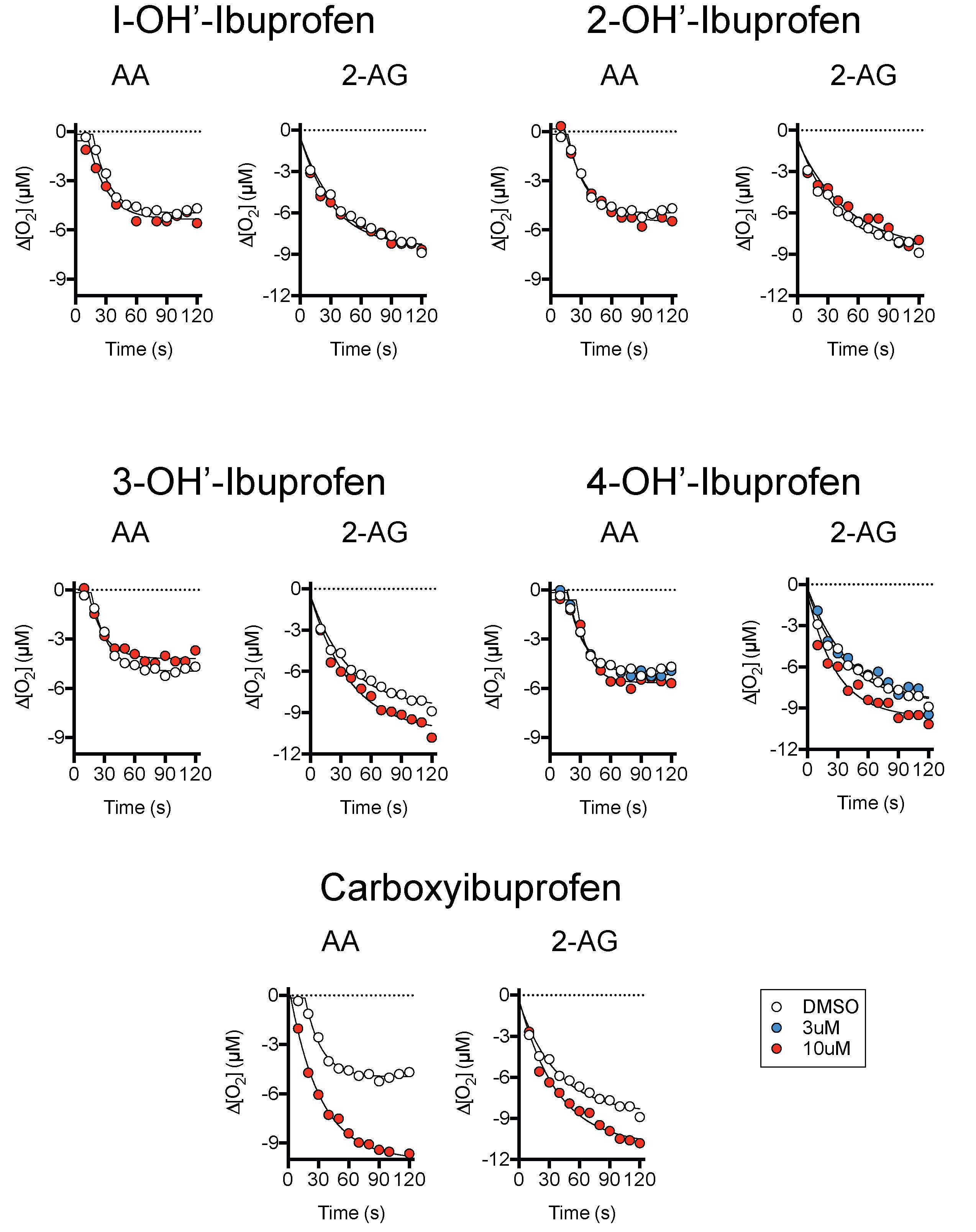

Supplement: Figure S2 — Inhibition of the activities of human recombinant COX-2 (towards either arachidonic acid or 2-AG) by the metabolites of ibuprofen. The substrates used (10 µM) are shown in the figure, as are the concentrations (in µM) of the test compounds. Values are means of two experiments of the change in oxygen utilisation following addition of enzyme to the oxygen electrode chamber. (TIF) [file pone.0103589.s002.tif]
